# Effects of extended-release niacin/laropiprant on correlations between apolipoprotein B, LDL-cholesterol and non-HDL-cholesterol in patients with type 2 diabetes

**DOI:** 10.1186/s12944-016-0282-8

**Published:** 2016-07-12

**Authors:** Eliot A. Brinton, Joseph Triscari, Philippe Brudi, Erluo Chen, Amy O. Johnson-Levonas, Christine McCrary Sisk, Rae Ann Ruck, Alexandra A. MacLean, Darbie Maccubbin, Yale B. Mitchel

**Affiliations:** Division of Atherometabolic Research, Utah Foundation for Biomedical Research, 420 Chipeta Way, Room 1160, Salt Lake City, UT 84108 USA; Merck & Co., Inc., Kenilworth, NJ USA

**Keywords:** Extended-release niacin/laropiprant, Type 2 diabetes mellitus, ApoB, LDL-C and non-HDL-C

## Abstract

**Background:**

LDL-C, non-HDL-C and ApoB levels are inter-correlated and all predict risk of atherosclerotic cardiovascular disease (ASCVD) in patients with type 2 diabetes mellitus (T2DM) and/or high TG. These levels are lowered by extended-release niacin (ERN), and changes in the ratios of these levels may affect ASCVD risk. This analysis examined the effects of extended-release niacin/laropiprant (ERN/LRPT) on the relationships between apoB:LDL-C and apoB:non-HDL-C in patients with T2DM.

**Methods:**

T2DM patients (*n* = 796) had LDL-C ≥1.55 and <2.97 mmol/L and TG <5.65 mmol/L following a 4-week, lipid-modifying run-in (~78 % taking statins). ApoB:LDL-C and apoB:non-HDL-C correlations were assessed after randomized (4:3), double-blind ERN/LRPT or placebo for 12 weeks. Pearson correlation coefficients between apoB:LDL-C and apoB:non-HDL-C were computed and simple linear regression models were fitted for apoB:LDL-C and apoB:non-HDL-C at baseline and Week 12, and the correlations between measured apoB and measured vs predicted values of LDL-C and non-HDL-C were studied.

**Results:**

LDL-C and especially non-HDL-C were well correlated with apoB at baseline, and treatment with ERN/LRPT increased these correlations, especially between LDL-C and apoB. Despite the tighter correlations, many patients who achieved non-HDL-C goal, and especially LDL-C goal, remained above apoB goal. There was a trend towards greater increases in these correlations in the higher TG subgroup, non-significant possibly due to the small number of subjects.

**Conclusions:**

ERN/LRPT treatment increased association of apoB with LDL-C and non-HDL-C in patients with T2DM. Lowering LDL-C, non-HDL-C and apoB with niacin has the potential to reduce coronary risk in patients with T2DM.

## Highlights

- LDL-C, ApoB and non-HDL-C are markers of coronary risk.
- Niacin reduces LDL-C, ApoB and non-HDL-C, with or without concurrent statin treatment.
- Statins increase the strength of correlations of ApoB with LDL-C and non-HDL-C.
- We studied the effects of niacin on these correlations in patients with diabetes.
- Niacin also increased the strength of correlations of ApoB with LDL-C and non-HDL-C.

## Background

The prevalence of type 2 diabetes mellitus (T2DM) is high. In 2010, an estimated 19.7 million Americans, 8.3 % of the adult population, had diagnosed T2DM [1]. An additional 8.2 million Americans had undiagnosed T2DM, and 38.2 % had pre-diabetes, with abnormal fasting glucose levels [1]. Patients with T2DM have a two- to four-fold elevated risk of cardiovascular disease relative to people without diabetes [2]. The dyslipidemia commonly associated with T2DM is typified by elevated plasma triglycerides (TG), low high-density lipoprotein cholesterol (HDL-C) levels, and a preponderance of small, dense low-density lipoprotein (LDL) particles [3].

Aggressive treatment of dyslipidemia is recommended for patients with T2DM to reduce coronary heart disease (CHD) risk, with the cornerstone of treatment being statin therapy. The Third Report of the National Cholesterol Education Program Expert Panel on Detection, Evaluation, and Treatment of High Blood Cholesterol in Adults (NCEP ATP-III) suggested treatment to achieve a low-density lipoprotein cholesterol (LDL-C) goal <2.59 mmol/L and optionally <1.81 mmol/L, with corresponding goals for non-high-density lipoprotein cholesterol (non-HDL-C) of <3.36 mmol/L and <2.59 mmol/L, respectively, in patients with the highest CHD risk [4–8]. In November 2013, the American College of Cardiology and American Heart Association (ACC/AHA) released cholesterol guidelines which did not endorse LDL-C and non-HDL-C treatment goals [9], but this deletion has been rejected by several professional national and international societies [10–12]. Further, it is important to note that the NHLBI, which had sponsored the lengthy data review and writing efforts (completely independent of the ACC and AHA), decided at the end of these processes that the resulting document should be published simply as an “evidentiary review.” [13] For this reason, it seems inappropriate that subsequent cursory ACC/AHA review and approval of the NHLBI-derived document be considered to have produced an official US guideline. Thus, primary reliance on lipid goals continues to be quite reasonable in clinical practice worldwide. Finally, despite the fact that the main lipid guideline emphasis traditionally has been on LDL-C, recent evidence suggests that apolipoprotein (apo) B and non-HDL-C may be better indicators of CHD risk, especially for patients with T2DM and/or elevated TG [14–19].

Many patients with T2DM fail to reach their LDL-C, non-HDL-C and apoB goals with statin therapy alone, in which case combination therapy with other lipid-modifying agents has been suggested [4, 5, 7, 8, 20]. Niacin (nicotinic acid) is a lipid-modifying agent that lowers LDL-C, apoB, non-HDL-C and TG levels and raises HDL-C levels. The combination of statins and niacin produces additive and complementary effects on plasma lipid/lipoprotein profiles. Previous clinical trials demonstrated that niacin monotherapy reduced myocardial infarction at 5 years and cardiovascular mortality at 15 years and, in combination with other lipid-modifying therapies (statins, bile acid resins, or both), slowed progression/induced regression of atherosclerotic plaque in patients with cardiovascular disease [21–24]. In contrast, two recent coronary outcomes trials failed to show that the addition of extended release niacin (ERN) to statin monotherapy further reduced cardiovascular events in patients with established cardiovascular disease and low baseline LDL-C levels, at least in the overall study populations [25, 26]. Subgroups with greater dyslipidemia, however, were noted to benefit [26, 27]. Further, niacin remains a treatment option for certain disease states and specific patient groups (e.g., statin-intolerant patients). Although niacin may not be used extensively for the treatment of patients with T2DM due to its tendency to worsen glycemic control, it is a reasonable choice in T2DM patients who are statin-intolerant and who have well-controlled plasma glucose levels.

### Objective

Several studies have evaluated the effects of statin therapy on the relationships (i.e., correlations and concordances) between apoB:LDL-C and apoB:non-HDL-C [28–34], and a recent study showed increasing strength in these correlations following treatment with either ERN/LRPT, a statin, or their combination [35]. A recent meta-analysis of statin trials found that on-treatment non-HDL-C and apoB and LDL-C all predicted subsequent cardiovascular events, and non-HDL-C was the best predictor among them [14]. The effect of ERN (with or without laropiprant) on apoB:LDL-C and apoB:non-HDL-C has not been investigated to date in patients with T2DM.

This study is a post-hoc analysis of a previously published clinical trial of ERN in combination with the flushing pathway inhibitor, laropiprant (ERN/LRPT), which significantly improved LDL-C, HDL-C and TG levels in patients with T2DM [36]. Although ERN/LRPT has been associated with a statistically significant increase in the incidence of certain non-fatal serious adverse events [26] and, therefore, has been withdrawn from the market and from further clinical development, laropiprant did not appear to alter the lipid effects of ERN. Thus, the data analyzed in this study are considered relevant to ongoing clinical use of ERN without laropiprant. This is the first study to compare the effects of ERN/LRPT treatment versus placebo (at 12 weeks) on the relationships between apoB:LDL-C and apoB:non-HDL-C in patients with T2DM. Given the known influence of elevated TG levels on LDL particle composition, these effects were further examined in patient subgroups with higher or lower baseline TG value, using a clinically meaningful cutoff of < and ≥2.26 mmol/L (200 mg/dL).

## Methods

### Study design

Post-hoc analysis was performed using data from a previously published, worldwide, multicenter (32 sites in the United States and 62 international sites), double-blind, randomized, placebo-controlled, parallel study of dyslipidemic patients with T2DM [36]. The study included a 4-week lipid-modifying run-in period followed by a 36-week double-blind treatment period. Complete details regarding study design and patient-entry criteria are published elsewhere (Protocol 069, Clinical Trials.gov: NCT00485758). The study protocol was approved by the institutional review boards at every study center and informed consent was obtained from each subject before the initiation of any study procedures. The study was conducted in accordance with the principles of Good Clinical Practice.

### Patients

Eligible patients included men and women, ages 18 to 80 years, with a diagnosis of T2DM, taking a stable dose of anti-diabetes medication for 3 months prior to study start. Patients had LDL-C ≥1.55 and <2.97 mmol/L (greater than or equal to 60 and less than 115 mg/dL) and TG <5.65 mmol/L (less than 500 mg/dL) following a 4-week, lipid-modifying run-in before the randomization visit. Approximately 78 % of patients were taking statins at baseline and were permitted to continue those medications during the study. Patients were excluded if they had the following laboratory values at Visit 1: creatinine >2.0 mg/dL, creatine kinase (CK) >2× the upper limit of normal (ULN), alanine aminotransferase (ALT), aspartate aminotransferase (AST) >1.5× ULN, or an abnormal thyroid stimulating hormone level (>20 % above the ULN). Other exclusion criteria included HbA1c >8.5 % at the screening visit or Visit 1, recent (new) diagnosis of T2DM or initiation of anti-obesity therapy within 3 months of Visit 1, use of systemic corticosteroids, and cyclical hormone contraceptives or other intermittent hormone therapies in female patients. Permitted lipid-altering therapies included dietary supplement omega-3, HMG-CoA reductase inhibitors (“statins”), fibrates (gemfibrozil, fenofibrate), ezetimibe, ezetimibe/simvastatin combination tablet, and bile-acid sequestrants. Patients taking therapies including niacin (>50 mg/day), Cholestin™, and fibrates in combination with a statin were excluded.

### Treatment

Patients were randomized 4:3 to ERN/LRPT 1 g/20 mg (1 tablet) or placebo. After 4 weeks of double-blind treatment, doses of active drug and placebo were doubled, increasing the ERN/LRPT to 2 g/40 mg (2 tablets) for the remainder of the study. No adjustments to background lipid-modifying regimens were made for the first 12 weeks of the study.

### Clinical laboratory measurements

A Center for Disease Control-certified laboratory conducted all clinical laboratory analyses using fasting blood samples. Total cholesterol (TC) and TG were measured by enzymatic methods. LDL-C was calculated by use of the Friedewald equation [37]. Non-HDL-C was calculated by subtracting HDL-C from TC values. ApoB was measured in whole plasma by radioimmunoassay and nephelometry.

### Statistical methods

This post-hoc analysis was performed on the subset of patients (90 %; 716/796) who had a baseline and Week 12 or later value for all three variables of interest (i.e., apoB, LDL-C and non-HDL-C). The statistical methods have been described previously in a similar analysis.^35^ In brief, the analyses were performed in a modified intent-to-treat population (*n* = 768) in the full analysis set (FAS) population at Week 12, including all randomized patients who had baseline and at least one post-Week 4 measurements, and had received at least one dose of study medication. Subgroup analyses were performed for patients defined by TG values, as follows: lower, <2.26 mmol/L (less than 200 mg/dL, normal to borderline-high TG) and higher, >2.26 mmol/L (hypertriglyceridemic).

An analysis of covariance (ANCOVA) model with terms for treatment, country, gender, and corresponding baseline lipid value as covariates was used to compare least squares (LS) mean percent changes from baseline in LDL-C, non-HDL-C and apoB between treatment groups. The placebo-subtracted differences in LS mean percent changes from baseline with 95 % confidence intervals (CI) were estimated from the ANCOVA model.

To examine the linear relationships at baseline and study end (week 12), simple linear regression models with apoB as a response variable were fitted on the overall population who had paired baseline and post-baseline values for apoB and LDL-C and apoB and non-HDL-C. The predicted values of LDL-C and non-HDL-C for known apoB values of 0.8 g/L or 0.9 g/L were calculated from the models. Pearson correlation coefficients between apoB:LDL-C and apoB:non-HDL-C were computed to inform the strength and direction of the correlations.

The degree of concordance between apoB:LDL-C and apoB:non-HDL-C were analyzed. Here, each patient was categorized into a quintile for apoB, LDL-C and non-HDL-C; those in the same quintile for the two parameters being compared were considered concordant, whereas those in different quintiles were considered discordant. The weighted kappa statistic was used to quantify the overall degree of concordance between the parameters. The concordance analyses were performed for the overall population and within each treatment group at baseline and endpoint; for each population, the concordance analyses were examined by baseline TG subgroup (i.e., < and ≥2.26 mmol/L, or 200 mg/dL).

## Results

Approximately 78 % of patients were taking a statin at baseline and continued to receive this treatment throughout the study. The baseline demographic and lipid/lipoprotein characteristics were generally well balanced between the ERN/LRPT and placebo groups in the overall population and within the patient subgroups defined by baseline TG value (Table 1). As expected, patients with higher versus lower baseline TG tended to have lower HDL-C levels and higher apoB, non-HDL-C and total cholesterol levels at baseline. Patients with higher versus lower baseline TG also tended to have higher fasting plasma glucose and glycosylated hemoglobin levels at baseline. Within the higher baseline TG subgroup, a slightly greater proportion of patients in the ERN/LRPT group were not taking a lipid-lowering medication at baseline compared with the placebo group.

**Table 1:** Baseline characteristics by treatment group

Parameter	ERN/LRPT			Placebo		
	(*N* = 393)			(*N* = 323)		
	Overall Population	Baseline TG subgroup		Overall Population	Baseline TG subgroup	
		<2.26 mmol/L (*n* = 320)	≥2.26 mmol/L (*n* = 73)		<2.26 mmol/L (*n* = 279)	≥2.26 mmol/L (*n* = 44)
Age, mean ± SD, y	61.80 ± 9.29	62.08 ± 9.27	60.58 ± 9.36	61.77 ± 9.43	62.04 ± 9.33	60.09 ± 9.99
Age, median (range), y	63 (31–79)	63 (33–79)	61 (31–76)	63 (21–80)	63 (21–80)	63 (36–77)
Sex, no. (%)						
Male	233 (59.3)	187 (58.4)	46 (63.0)	208 (64.4)	182 (65.2)	26 (59.1)
Female	160 (40.7)	133 (41.6)	27 (37.0)	115 (35.6)	97 (34.8)	18 (40.9)
Population subgroups, no. (%)^a^						
Statin-treated	310 (78.9)	259 (80.9)	51 (69.9)	259 (80.2)	227 (81.4)	32 (72.7)
Other LMT-treated	33 (8.4)	24 (7.5)	9 (12.3)	34 (10.5)	28 (10.0)	6 (13.6)
Drug naive	72 (18.3)	54 (16.9)	18 (24.7)	55 (17.0)	47 (16.8)	8 (18.2)
BMI, mean ± SD, kg/m^2^	31.28 ± 6.45	30.90 ± 6.43	32.93 ± 6.34	30.48 ± 5.83	30.26 ± 5.92	31.84 ± 5.01
Baseline values, mean ± SD						
apoB, g/L	0.93 ± 0.19	0.90 ± 0.17	1.1 ± 0.18	0.91 ± 0.17	0.89 ± 0.15	1.1 ± 0.20
TC, mmol/L	4.30 ± 0.68	4.20 ± 0.67	4.69 ± 0.58	4.21 ± 0.64	4.14 ± 0.60	4.65 ± 0.75
Non-HDL-C, mmol/L	3.00 ± 0.64	2.85 ± 0.57	3.64 ± 0.54	2.91 ± 0.58	2.81 ± 0.50	3.53 ± 0.64
HDL-C, mmol/L	1.29 ± 0.36	1.35 ± 0.35	1.04 ± 0.25	1.30 ± 0.34	1.33 ± 0.35	1.12 ± 0.25
LDL-C, mmol/L	2.26 ± 0.53	2.26 ± 0.53	2.27 ± 0.55	2.20 ± 0.47	2.19 ± 0.44	2.27 ± 0.61
TG^b^, mmol/L	1.42 (0.36–7.10)	1.27 (0.36–2.23)	2.88 (2.28–7.10)	1.45 (0.44–5.75)	1.32 (0.44–2.24)	2.63(2.26–5.75)
CRP^b^, mg/L	1.8 (0.1–56.2)	1.7 (0.1–56.2)	2.3 (0.3–36.3)	1.5 (0.1–69.7)	1.5 (0.1–69.7)	1.40 (0.3–15.7)
FPG, mmol/L	7.29 ± 1.81	7.10 ± 1.63	8.11 ± 2.28	7.41 ± 1.79	7.38 ± 1.83	7.62 ± 1.59
HbA1c, %	6.91 ± 0.72	6.86 ± 0.69	7.11 ± 0.81	6.90 ± 0.70	6.89 ± 0.69	6.97 ± 0.74

*ApoB* apolipoprotein B; *BMI* body mass index; *ERN/LRPT* ER niacin/laropiprant; *HDL-C* high-density lipoprotein cholesterol; *LDL-C* low-density lipoprotein cholesterol; *TC* total cholesterol; *TG* triglyceride; *CRP* C-reactive protein; *FPG* fasting plasma glucose; *HbA1c* hemoglobin A1c

^a^Because a patient can be taking both a statin and another LMT at baseline, the total percentage may be greater than 100 %

^b^Expressed as median

**Fig. 1:**
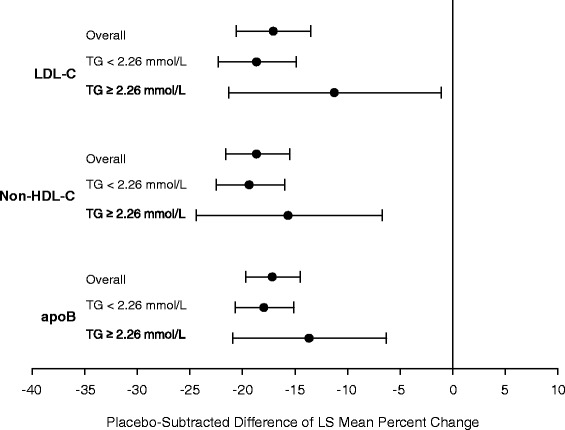
Between-group differences (with 95 % confidence interval) in least square (LS) mean percentage changes from baseline at Week 12 in LDL-C, non-HDL-C and apoB for the overall population and patient subgroups defined by baseline triglyceride (TG) value (i.e., < and ≥2.26 mmol/L)

In the overall population and within patient subgroups defined by baseline TG, treatment with ERN/LRPT significantly reduced LDL-C, non-HDL-C and apoB versus placebo at Week 12 (Fig. 1). Similar placebo-adjusted reductions of approximately 17, 19 and 17 % were observed for LDL-C, non-HDL-C and apoB, respectively, following treatment with ERN/LRPT in the overall population. The placebo-adjusted decreases in LDL-C, non-HDL-C and apoB were numerically, but not significantly, smaller with ERN/LRPT in the higher versus lower TG subgroup.

**Fig. 2:**
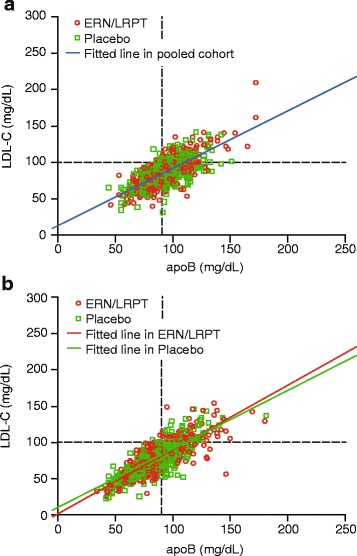
Scatterplots of apoB versus LDL-C at baseline (**a**) and following treatment with ERN/LRPT or placebo for 12 weeks (**b**). The upper thresholds for the less-stringent LDL-C <100 mg/dL and apoB <90 mg/dL goals are denoted by horizontal and vertical lines, respectively. Right lower quadrant shows the large number of subjects who met LDL-C goal <100 mg/dL but did not reach apoB goal <90 mg/dL after niacin treatment

**Fig. 3:**
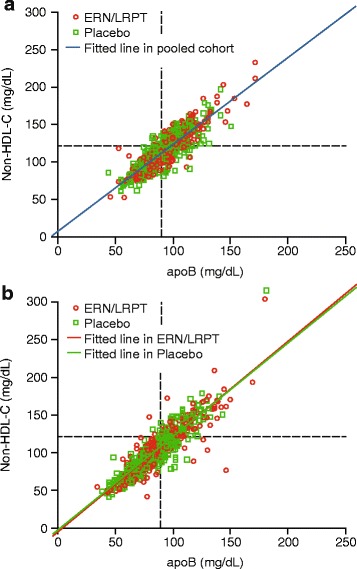
Scatterplots of apoB versus non-HDL-C at baseline (**a**) and following treatment with ERN/LRPT or placebo at Week 12 (**b**). The upper thresholds for the less-stringent non-HDL-C <130 mg/dL and apoB <90 mg/dL goals are denoted by horizontal and vertical lines, respectively. Right lower quadrant shows the large number of subjects who met non-HDL-C goal <130 mg/dL but did not reach apoB goal <90 mg/dL after niacin treatment

Strong positive correlations between apoB:LDL-C and apoB:non-HDL-C were observed at baseline and following treatment with ERN/LRPT or placebo, irrespective of baseline TG level (Table 2; Figs. 2 and 3). Compared with LDL-C, non-HDL-C was more highly correlated with apoB at baseline and Week 12 in the overall population and in both patient subgroups defined by baseline TG. Treatment with ERN/LRPT increased the strength of the correlations between apoB and LDL-C in the overall group and tended to increase the correlations in both lower and high TG groups. ERN/LRPT produced less pronounced increases in the correlations between apoB and non-HDL-C, although the on-treatment correlations were higher than those for apoB and LDL-C (Table 2; Figs. 2 and 3). The improvements in the apoB:LDL-C and apoB:non-HDL-C correlations with ERN/LRPT treatment tended to be greater in the overall population and in the higher versus the lower TG subgroup. The correlations between apoB:LDL-C and apoB:non-HDL-C were generally similar at baseline and study end in the placebo group for the overall population and within both TG subgroups.

**Table 2:** Slope, intercept, Pearson correlation coefficient, and predicted LDL-C and non-HDL-C values based on simple linear regression of LDL-C or non-HDL-C on apoB at baseline (i.e., prior to receiving randomized treatment) and Week 12

Treatment Group	N^a^	Slope	Intercept	apoB vs LDL-C Pearson Correlation Coefficient (95 % CI) [R^2^]	Predicted^b^ LDL-C Value (95 % CI) given apoB of 0.8 g/L	Predicted^b^ LDL-C Value (95 % CI) given apoB of 0.9 g/L
Baseline (i.e., pre-treatment measurement)						
Pooled across treatment groups	721	2.04	0.36	0.72 (069, 0.76) [0.53]	1.98 (1.95, 2.01)	2.18 (2.16, 2.21)
TG <2.26 mmol/L	602	2.35	0.13	0.77 (0.74, 0.80) [0.59]	2.01 (1.98, 2.04)	2.24 (2.22, 2.27)
TG ≥2.26 mmol/L	119	2.35	−0.27	0.78 (0.70, 0.84) [0.61]	1.61 (1.50, 1.73)	1.85 (1.76, 1.93)
ERN/LRPT	395	2.04	0.35	0.73 (0.68, 0.77) [0.53]	1.99 (1.94, 2.03)	2.19 (2.15, 2.23)
TG <2.26 mmol/L	322	2.43	0.07	0.80 (0.75, 0.83) [0.64]	2.02 (1.98, 2.06)	2.26 (2.23, 2.30)
TG ≥2.26 mmol/L	73	2.22	−0.16	0.74 (0.61, 0.83) [0.55]	1.63 (1.47, 1.79)	1.85 (1.73, 1.98)
Placebo	326	1.99	0.38	0.72 (0.66, 0.76) [0.51]	1.98 (1.93, 2.02)	2.18 (2.14, 2.21)
TG <2.26 mmol/L	280	2.20	0.23	0.72 (0.66, 0.78) [0.53]	2.00 (1.96, 2.04)	2.22 (2.18, 2.26)
TG ≥2.26 mmol/L	46	2.51	−0.41	0.85 (0.73, 0.91) [0.72]	1.59 (1.43, 1.75)	1.84 (1.71, 1.97)
Week 12						
ERN/LRPT	394	2.30	0.04	0.82 (0.79, 0.85) [0.68]	1.88 (1.84, 1.91)	2.11 (2.07, 2.15)
TG <2.26 mmol/L	321	2.61	−0.16	0.84 (0.80, 0.87) [0.70]	1.93 (1.90, 1.97)	2.20 (2.15, 2.24)
TG ≥2.26 mmol/L	73	1.99	0.13	0.86 (0.78, 0.91) [0.74]	1.73 (1.65, 1.81)	1.93 (1.86, 2.00)
Placebo	323	2.09	0.27	0.73 (0.68, 0.78) [0.54]	1.94 (1.89, 1.98)	2.14 (2.07, 2.15)
TG <2.26 mmol/L	279	2.35	0.06	0.77 (0.71, 0.81) [0.59]	1.94 (1.89, 1.98)	2.17 (2.13, 2.21)
TG ≥2.26 mmol/L	44	1.68	0.50	0.69 (0.49, 0.82) [0.47]	1.84 (1.66, 2.02)	2.01 (1.86, 2.15)
Treatment Group	N^a^	Slope	Intercept	apoB vs non-HDL-C Pearson Correlation Coefficient (95 % CI) [R2]	Predicted^b^ Non-HDL-C Value (95 % CI) given apoB of 0.8 g/L	Predicted^b^ Non-HDL-C Value (95 % CI) given apoB of 0.9 g/L
Baseline (i.e., pre-treatment measurement)						
Pooled across treatment groups	721	2.97	0.21	0.87 (0.85, 0.89) [0.76]	2.59 (2.56, 2.62)	2.89 (2.87, 2.91)
TG <2.26 mmol/L	602	2.82	0.31	0.84 (0.82, 0.87) [0.71]	2.57 (2.54, 2.60)	2.85 (2.83, 2.88)
TG ≥2.26 mmol/L	119	2.59	0.81	0.85 (0.79, 0.89) [0.72]	2.88 (2.78, 2.98)	3.14 (3.06, 3.21)
ERN/LRPT	395	2.95	0.25	0.87 (0.85, 0.89) [0.76]	2.61 (2.57, 2.65)	2.90 (2.87, 2.94)
TG <2.26 mmol/L	322	2.82	0.32	0.86 (0.83, 0.89) [0.74]	2.58 (2.54, 2.61)	2.86 (2.83, 2.89)
TG ≥2.26 mmol/L	73	2.38	1.07	0.81 (0.71, 0.87) [0.65]	2.97 (2.83, 3.10)	3.20 (3.10, 3.31)
Placebo	326	3.00	0.17	0.87 (0.84, 0.89) [0.75]	2.57 (2.53, 2.61)	2.87(2.84, 2.90)
TG <2.26 mmol/L	280	2.82	0.30	0.82 (0.78, 0.86) [0.67]	2.56 (2.52, 2.60)	2.84 (2.81, 2.88)
TG ≥2.26 mmol/L	46	2.87	0.46	0.91 (0.83, 0.95) [0.82]	2.76 (2.62, 2.90)	3.05 (2.94, 3.15)
Week 12						
ERN/LRPT	394	3.28	-0.13	0.89 (0.87, 0.91) [0.79]	2.49 (2.45, 2.53)	2.82 (2.78, 2.86)
TG <2.26 mmol/L	321	3.15	-0.06	0.87 (0.84, 0.90) [0.76]	2.47 (2.43, 2.51)	2.78 (2.74, 2.83)
TG ≥2.26 mmol/L	73	3.36	-0.12	0.88 (0.82, 0.92) [0.78]	2.56 (2.44, 2.68)	2.89 (2.78, 3.00)
Placebo	323	3.21	-0.04	0.87 (0.84, 0.90) [0.76]	2.52 (2.48, 2.56)	2.84 (2.80, 2.88)
TG <2.26 mmol/L	279	3.00	0.12	0.85 (0.82, 0.88) [0.73]	2.52 (2.48, 2.57)	2.82 (2.79, 2.86)
TG ≥2.26 mmol/L	44	3.60	-0.33	0.89 (0.79, 0.93) [0.78]	2.55 (2.35, 2.75)	2.91 (2.75, 3.07)

*apoB* apolipoprotein B; *LDL-C* low-density lipoprotein cholesterol; *HDL-C* high-density lipoprotein cholesterol; *ERN/LRPT* niacin/lariproprant; *TG* triglyceride

^a^Number of patients with paired apoB and LDL-C or non-HDL-C measurements

^b^Predicted LDL-C or non-HDL-C value (mmol/L) assuming apoB value of 0.8 or 0.9 g/L

At baseline and Week 12, the predicted LDL-C and non-HDL-C values for known apoB values were below the targets of 2.59 mmol/L and 3.36 mmol/L, respectively (Table 2). When the regression analyses were examined by baseline TG level, the predicted LDL-C values were significantly lower in the high TG versus the low TG subgroup (i.e., 95 % CIs did not overlap) at baseline and Week 12. This finding was observed in both treatment groups, except in the placebo group at Week 12. In contrast, the predicted non-HDL-C values were significantly greater in the higher versus the lower TG subgroup at baseline, but not at Week 12.

Weighted kappa statistics to assess the degree of concordance between apoB:LDL-C and apoB:non-HDL-C before and after treatment with ERN/LRPT or placebo are presented in Table 3. A kappa value of 1 represents perfect overlap and a value of 0 represents a complete lack of overlap between 2 parameters. In general, the correlations assessed by Pearson correlation coefficients were corroborated with those assessed by the weighted kappa. For apoB:LDL-C and apoB:non-HDL-C, the weighted kappa values were generally similar between the treatment groups and TG subgroups at baseline. For both treatment groups and both TG subgroups, the weighted kappa values were higher for the comparison of apoB:non-HDL-C versus apoB:LDL-C at baseline and at study end. At baseline, the degree of concordance between apoB and LDL-C was moderate, while the degree of concordance between apoB and non-HDL-C was more substantial. At Week 12, treatment with ERN/LRPT generally increased the level of concordance more between apoB and LDL-C than between apoB and non-HDL-C relative to baseline and placebo.

**Table 3:** Degree of concordance among apoB, LDL-C, and non-HDL-C levels at baseline and study endpoint in the pooled treatment groups for the overall population and treatment subgroups defined by baseline TG < and ≥2.26 mmol/L

	N	Concordance between apoB and LDL-C	N	Concordance between apoB and Non-HDL-C
		Weighted kappa^a^ (95 % CI)		Weighted kappa^a^ (95 % CI)
Baseline (i.e., drug-naïve patients)				
Overall Population				
All Treatments	716	0.49 (0.44, 0.53)	716	0.66 (0.62, 0.69)
TG <2.26 mmol/L	599	0.52 (0.47, 0.56)	599	0.63 (0.60, 0.67)
TG ≥2.26 mmol/L	117	0.56 (0.46, 0.65)	117	0.64 (0.56, 0.72)
ERN/LRPT	393	0.50 (0.45, 0.56)	393	0.67 (0.63, 0.72)
TG <2.26 mmol/L	320	0.54 (0.49, 0.60)	320	0.65 (0.60, 0.70)
TG ≥2.26 mmol/L	73	0.56 (0.44, 0.68)	73	0.65 (0.56, 0.75)
Placebo	323	0.46 (0.40, 0.53)	323	0.65 (0.60, 0.70)
TG <2.26 mmol/L	279	0.50 (0.43, 0.57)	279	0.64 (0.58, 0.69)
TG ≥2.26 mmol/L	44	0.55 (0.39, 0.71)	44	0.71 (0.56, 0.85)
Endpoint (i.e., following randomized treatment)				
Overall Population				
All Treatments	716	0.61 (0.57, 0.64)	716	0.73 (0.70, 0.76)
TG <2.26 mmol/L	599	0.64 (0.60, 0.68)	599	0.73 (0.70, 0.76)
TG ≥2.26 mmol/L	117	0.57 (0.47, 0.67)	117	0.70 (0.63, 0.78)
ERN/LRPT	393	0.64 (0.60, 0.69)	393	0.73 (0.69, 0.77)
TG <2.26 mmol/L	320	0.66 (0.61, 0.71)	320	0.72 (0.67, 0.76)
TG ≥2.26 mmol/L	73	0.64 (0.54, 0.75)	73	0.71 (0.60, 0.82)
Placebo	323	0.53 (0.46, 0.59)	323	0.65 (0.60, 0.71)
TG <2.26 mmol/L	279	0.55 (0.49, 0.62)	279	0.66 (0.61, 0.72)
TG ≥2.26 mmol/L	44	0.53 (0.38, 0.69)	44	0.68 (0.56, 0.81)

*ERN/LRPT* niacin/laropiprant; *apoB* apolipoprotein *B* DL-C low-density lipoprotein cholesterol; *HDL-C* high-density lipoprotein cholesterol; *TG* triglyceride

^a^The k statistic, on a scale from 0 to 1, reflects the degree of agreement between two variables. The levels of agreement range from slight (0–0.20), fair (0.21–0.40), moderate (0.41–0.60), substantial (0.61–0.80), and almost perfect (0.81–1.00)

## Discussion

The influence of statin therapy on the correlations between apoB:LDL-C and apoB:non-HDL-C in dyslipidemic patients has been well studied [30–34]. Statin therapy produces smaller percentage reductions in apoB than in LDL-C and non-HDL-C. Further, statin therapy strengthens the linear relationships between apoB:LDL-C and apoB:non-HDL-C, as evidenced by increases in the correlation coefficients and kappa values (i.e., a measure of the degree of concordance between 2 parameters) for these parameters in statin-treated versus untreated patients. Although the influence of non-statin lipid-modifying drugs on the linear relationships between these lipid/lipoprotein parameters has been less well studied, a recent analysis evaluated the effects of ERN/LRPT, simvastatin (SIMVA), and ERN/LRPT + SIMVA on apoB:LDL-C and apoB:non–HDL-C correlations in patients with dyslipidemia [35]. The results demonstrated that both LDL-C and non–HDL-C were well correlated with apoB at baseline and following treatment with ERN/LRPT, SIMVA and the combination of both, and the correlations were higher and the predicted LDL-C and non–HDL-C levels based on apoB were considerably lower compared with baseline [35].

The primary purpose of the present analysis was to examine the effects of ERN/LRPT on the relationships (i.e., correlation coefficients, linear regression analyses and kappa values) between apoB:LDL-C and apoB:non-HDL-C in patients with T2DM. At baseline, approximately 78 % of patients were receiving statin-based (non-niacin) lipid-modifying therapy and were permitted to continue these medications throughout the study. Thus, these analyses examine the effects of ERN/LRPT on relationships between apoB:LDL-C and apoB:non-HDL-C in T2DM patients, the majority of whom were taking a statin-based therapy. Given the known influence of TG levels on LDL particle composition, the relationships between these lipid/lipoprotein parameters were also examined at baseline and following treatment in subgroups of patients with lower and higher baseline TG values.

Treatment with ERN/LRPT produced similar magnitude reductions from baseline in apoB, LDL-C and non-HDL-C in patients with T2DM (i.e., mean reductions ranging from 17 to 19 % across the lipid/lipoprotein parameters). A trend toward smaller magnitude reductions in apoB, LDL-C and non-HDL-C were observed in patients with higher versus lower baseline TG values (> and <2.26 mmol/L). Nevertheless, similar magnitude mean percent reductions from baseline in apoB, LDL-C and non-HDL-C were observed when the treatment effects were examined separately within each of the TG subgroups. Overall, the findings of the current study support prior study results, showing that treatment with ERN/LRPT and ERN (without LRPT) produces somewhat comparable reductions in apoB, LDL-C and non-HDL-C in patients with dyslipidemia [38]. This study, for the first time, extends these findings to include patients with T2DM, irrespective of baseline TG level. In contrast, studies of statin monotherapy show significantly larger magnitude reductions in LDL-C and non-HDL-C versus apoB in a wide variety of patient populations, including those with T2DM and hypertriglyceridemia [19, 28]. The differential effects of ERN compared with statins on apoB, LDL-C and non-HDL-C are likely due to differences in the mechanisms of actions of these agents.

This analysis demonstrated strong, linear relationships between apoB:LDL-C and apoB:non-HDL-C, both at baseline and following treatment with ERN/LRPT. Treatment with ERN/LRPT increased the strengths of the correlation coefficients between apoB:LDL-C, with a less pronounced effect on apoB:non-HDL-C. The strengths of the correlations between apoB:LDL-C and apoB:non-HDL-C were generally stronger in the higher versus the lower TG subgroup.

For the overall population, the predicted LDL-C and non-HDL-C levels corresponding to known apoB values at baseline were already below the targets of 2.59 mmol/L and 3.36 mmol/L, respectively, and these levels were further lowered following treatment with ERN/LRPT. When examined by TG subgroup, the predicted LDL-C values were lower in the higher TG versus the lower TG subgroup, both at baseline and study endpoint. Conversely, the predicted non-HDL-C values were higher in the high TG versus the lower TG subgroup at baseline, but this finding was less pronounced at study endpoint.

ApoB was more concordant with non-HDL-C than with LDL-C, both at baseline and study endpoint, irrespective of the TG subgroup. This was expected since apoB is a measure of the number of non-HDL particles. Treatment with ERN/LRPT increased the concordance between apoB:non-HDL-C less than that between apoB:LDL-C, although the former remained higher than the latter.

## Conclusion

In conclusion, the apoB:LDL-C and apoB:non–HDL-C correlation coefficients and kappa values were higher and the predicted LDL-C and non–HDL-C levels for a known apoB value were lower following treatment with ERN/LRPT compared with baseline levels. Interestingly, this was despite the fact that there were greater percent decreases in LDL-C and non-HDL-C than in apoB. Although some discordance remains between non-HDL-C and apoB levels, these data suggest that there may be only modest benefit from measurement of apoB in addition to the non-HDL-C level available as part of the standard lipid panel. These data also show the attainment of more aggressive LDL-C and non-HDL-C goals in patients receiving ERN in combination with a statin, reminding of the potential of combination lipid-modifying therapy for further reduction of coronary risk by helping to normalize apoB-containing atherogenic lipoprotein composition and levels.

## Abbreviations

apoB: apolipoprotein B
apoB:LDL-C: apoB and LDL-C
apoB:non-HDL-C: apoB and non-HDL-C
ASCVD: atherosclerotic cardiovascular disease
CHD: coronary heart disease
ERN/LRPT: extended-release niacin/laropiprant fixed-dose combination
HDL-C: high-density lipoprotein cholesterol
LDL-C: low-density lipoprotein cholesterol
T2DM: type 2 diabetes mellitus
TG: triglyceride (s)
